# Infection control and the burden of tuberculosis infection and disease in health care workers in china: a cross-sectional study

**DOI:** 10.1186/1471-2334-10-313

**Published:** 2010-10-28

**Authors:** Guang Xue He, Susan van den Hof, Marieke J van der Werf, Guo Jie Wang, Shi Wen Ma, Dong Yang Zhao, Yuan Lian Hu, Shi Cheng Yu, Martien W Borgdorff

**Affiliations:** 1 National Center for TB control and prevention, China Center for Disease Control and Prevention (CDC), Changping District 102206, Beijing, China; 2 Center for Infection and Immunity Amsterdam (CINIMA), Academic Medical Center, University of Amsterdam, Meibergdreef 9, 1105 AZ, Amsterdam, The Netherlands; 3 KNCV Tuberculosis Foundation, Parkstraat 17, 2514 JD, The Hague, The Netherlands; 4 Tuberculosis Control Institute, Henan Provincial Center for Disease Control and Prevention, Xindong Districe 450016, Zhengzhou, China; 5 National Center for Public Health Surveillance and Information Service, Changping District 102206, China CDC, Beijing, China

## Abstract

**Background:**

Hospitals with inadequate infection control are risky environments for the emergence and transmission of tuberculosis (TB). We evaluated TB infection control practices, and the prevalence of latent TB infection (LTBI) and TB disease and risk factors in health care workers (HCW) in TB centers in Henan province in China.

**Methods:**

A cross-sectional survey was conducted in 2005. To assess TB infection control practices in TB centers, checklists were used. HCW were tuberculin skin tested (TST) to measure LTBI prevalence, and were asked for sputum smears and chest X-rays to detect TB disease, and questionnaires to assess risk factors. Differences between groups for categorical variables were analyzed by binary logistic regression. The clustered design of the study was taken into account by using a multilevel logistic model.

**Results:**

The assessment of infection control practices showed that only in a minority of the centers the patient consultation areas and X-ray areas were separated from the waiting areas and administrative areas. Mechanical ventilation was not available in any of the TB centers. N95 respirators were not available for HCW and surgical masks were not available for TB patients and suspects. The LTBI prevalence of HCW with and without BCG scar was 55.6% (432/777) and 49.0% (674/1376), respectively (P = 0.003). Older HCW, HCW with longer duration of employment, and HCW who worked in departments with increased contact with TB patients had a higher prevalence of LTBI. HCW who work in TB centers at the prefecture level, or with an inpatient ward also had a higher prevalence of LTBI. Twenty cases of pulmonary TB were detected among 3746 HCW. The TB prevalence was 6.7/1000 among medical staff and 2.5/1000 among administrative/logistic staff.

**Conclusion:**

TB infection control in TB centers in Henan, China, appears to be inadequate and the prevalence of LTBI and TB disease among HCW was high. TB infection control practices in TB centers should be strengthened in China, including administrative measures, renovation of buildings, and use of respirators and masks. Regular screening of HCW for TB disease and LTBI needs to be considered, offering preventive therapy to those with TST conversions.

## Background

China has the second largest number of tuberculosis (TB) cases in the world [1], and also is one of the countries with high levels of drug resistant TB [2]. Based on the data of the 4^th ^national TB epidemiological survey in 2000 [3], it is estimated that there were 4.5 million prevalent TB cases in China, of which 1.96 million were pulmonary, bacteriologically confirmed cases. Based on a recent national anti-tuberculosis drug resistance survey, it was estimated that approximately 120,000 new multi-drug resistant (MDR) TB cases emerge annually in China, including 9,000 extensively drug-resistant TB (XDR-TB) cases, accounting for approximately 24% of the global burden of MDR-TB [4].

Hospitals with inadequate infection control are risky environments for the emergence and transmission of respiratory infectious diseases, such as TB [5,6]. Multiple studies have documented the risk of TB transmission from patients to health care workers (HCW) and from patients to patients in low, medium, and high resource settings [7,8]. A diverse number of risk factors have been identified, most of which are related to prolonged, unprotected exposure to inpatients with untreated TB [7,8]. HCW surveillance, combined with administrative measures and appropriate exposure-based use of personal protective materials and environmental equipment, has helped reduce transmission of TB in some hospitals [9].

In most of the world, respiratory infection control in health care facilities remains inadequate [10]. A health care facility in South Africa was implicated as the initial site of transmission for the first documented outbreak of MDR-TB [11]. Earlier, inadequate infection control in hospitals had already led to large outbreaks of MDR-TB [12]. These outbreaks have turned attention to the need to reduce TB transmission in health care facilities [2]. New World Health Organization (WHO) guidelines on TB infection control have been released in 2009 and call on countries to institute programs to screen for TB regularly in HCW and to routinely record and report this data [10]. Health care facilities in China do not routinely screen HCW for latent TB infection (LTBI) and TB disease. Until recently, there was no national TB infection control policy, and also no occupational TB policy in place in China. However, the high number of MDR-TB cases in China, many of whom receive prolonged treatment in hospitals, adds to the importance of screening HCW and preventing nosocomial transmission of (MDR-) TB.

In order to measure the prevalence of LTBI and TB disease in Chinese HCW, we conducted a study in Henan province, the province in China with the largest population size (97 million) and a high proportion of MDR-TB cases (12.9% in 2001 [13]). We assessed TB infection control practices in the TB centers, and the prevalence of LTBI and TB disease in its HCW. This would be the first study documenting the prevalence of LTBI and TB disease among HCW in China in the international scientific literature.

## Methods

### Research methods

#### Study design, population and sampling

**Figure 1:**
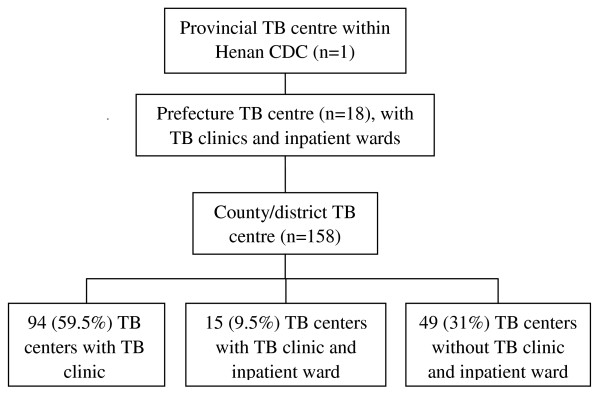
**Illustration of TB control system in Henan province**.

We conducted a cross-sectional survey on HCW from TB centers in Henan province, China. Henan province is divided into 18 prefecture-level administrative areas and the prefectures into 158 counties/districts. In each administrative level in Henan province, there is one TB center that is responsible for implementing its respective TB program. However, not every TB center has its own TB clinic. Each prefecture level and 109 (69%) of county/district level TB centers have TB clinics, while 49 of them do not. Of those with a TB clinic, 33 (26%) TB centers had an inpatient TB ward; 18 (100%) at prefecture level and 15 (14%) at county level (Figure 1).

All 127 TB centers with TB clinics were included in the survey on TB infection control practices and prevalence of TB disease among HCW. For the survey on LTBI, all 18 TB centers at the prefecture level and 40 randomly selected TB centers at the county/district level were included.

#### Data collection

The provincial level TB center staff conducted a pilot study in Zhumadian city in December 2004. The data are included in the study results. Provincial level staff trained prefecture level project staff in January 2005. Prefecture project staff then trained county/district project staff. The field investigation was conducted from January 17 to March 31, 2005.

Project staff from prefecture and county/district level filled in a checklist on prevailing TB infection control practices in their own center. To assess TB disease prevalence, chest X-rays were done on all HCW after they provided informed consent. Three sputum smears (spot, morning, evening) were examined following the WHO standard for HCW with an abnormal chest radiograph or cough longer than two weeks. All participants were asked to fill in a questionnaire on demographic characteristics and TB symptoms at the time of their X-ray examinations.

A TST was done on all HCW in the subset of 58 facilities, unless they declined to participate or were not available during the study period. In China, BCG vaccination is highly recommended by health authorities since the late 1970s and is usually given to all newborns within the first few days of life. No booster vaccinations are recommended in China. Therefore, the influence of BCG on the TST result is expected to be minor [8,14].

TST were conducted according to WHO procedures [15]. Chinese-made purified-protein derivative (PPD), 0.1 ml, 5 Tuberculin Unit was injected subcutaneously on the volar surface of the left forearm and the induration measured at 48-72 hours. Studies in China showed that the purity of the local PPD was similar to the international standard PPD (PPD-RT23) [16]. In direct comparison studies stability, safety, reaction size, and conversion rate also were similar [17,18]. TSTs were administered by 18 nurses who have attended a provincial training course and passed a qualification exam set up for this study. Each participant in the investigation was carefully examined for the presence of a Bacille de Calmette Guerin (BCG) scar before testing in order to ascertain the participant's vaccination status. Similar to other studies, we used TST induration ≥ 10 mm as a cut-off point [19-21], and defined the diagnosis of LTBI as a TST result with an induration ≥ 10 mm [7,22].

#### Quality control

In order to implement the survey successfully, provincial coordination and technical groups were established. The coordination group was responsible for organizing and coordinating the implementation of the study. The technical group was responsible for the training of prefecture level project staff, quality control, and checking and collecting all data. A research group was established in each prefecture and was responsible for organizing the field work and for quality control. The research group included TB control staff, TST technicians, and a deputy director of a TB center as the head of the group.

The provincial level technical group personnel and principal investigators reviewed all data collected each day to ensure that data were recorded legibly, completely, and consistently. The provincial technical group also conducted site visits to ensure quality of the data and sputum collection and examination.

#### Data analysis

Data were double entered using Epi Data 3.1 (The Epi Data Association, Odense, Denmark, 2003-2008) and checked for discrepancies between the two entries. Differences between groups for the distribution of categorical variables were analyzed by binary logistic regression. Odds ratios (OR) are presented with their 95% confidence intervals (CI).

The TST data showed terminal-digit preference. To smooth the data, we applied a five-point moving average. We compared TST results in HCW with and without BCG scar. The association of TST results with variables of interest was examined using a logistic regression model. We took the clustered design of the study into account by using a multilevel logistic model with two levels: the TB center and the individual staff members within the TB centers. Variables collected at the TB center level were prefecture versus county/district level, size of the TB center as measured by number of staff employed, and presence of an inpatient ward. Variables collected at the individual level included sex, age, position, job location, education, years of employment in this TB center, income, smoking and alcohol use. The test for the clustering effects showed a significant difference among TB centers, so a random effect logistic regression model was used. *MLwiN Version 2.02 *(Rasbash, J., Charlton, C., Browne, W.J., Healy, M. and Cameron, B. (2005) *MLwiN Version 2.02*. Centre for Multilevel Modeling, University of Bristol) was used for fitting the model.

#### Ethical issues

The research project was approved by the Chinese Ethical Committee for TB Operational Research in Beijing. The research staff provided HCW with basic background information about the study. Participants were then asked to read documentation themselves and provide written informed consent. The participants were informed of their sputum smear, chest X-ray, and TST results. All persons identified as having TB disease were advised to start treatment at the local TB center. Treatment for LTBI is not routinely offered in China. Those with positive TST results were informed that they should pay particular attention to TB symptoms because they are at higher risk of developing active TB.

## Results

### TB infection control situation

Of the 127 TB centers, 33 (26.0%) had pre-employment screening of HCW for TB disease in place, and 62 (48.8%) had a screening program for HCW that included annual screening using X-ray and physical examination during employment. The patient consultation areas and X-ray areas were in different rooms than the waiting areas and administrative areas in a minority of the TB centers (Table 1). Mechanical ventilation was not available in any TB center. TB center staff reported that windows were often opened in 43%-67% of patient consultation rooms, sputum examination rooms and X-ray rooms. 116 (91.3%) consultation rooms, 121 (95.3%) sputum examination rooms and 73 (57.5%) X-ray rooms had ultraviolet (UV) lights that were situated at upper room. In rooms with UV lights, the UV lights were used daily in 71 (61.2%) patient consultation rooms, 80 (66.1%) sputum examination rooms and 33 (45.2%) X-ray rooms. Only a minority of UV lights was tested regularly, and less than half of them were cleaned monthly. N95 respirators were not available for HCW and surgical masks were not available for TB patients and suspects.

**Table 1:** Tuberculosis infection control situation in 127 tuberculosis centers

	Separated from waiting room n (%)	Separated from administrative area. n (%)	Open windows often* n (%)	UVGI			
				
				Available n (%)	Used daily n (%)	Tested Regularly** n (%)	Cleaned monthly n (%)
Patient consultation room	14 (11.0)	18 (14.2)	83 (65.4)	116 (91.3)	71 (61.2)	13 (11.2)	57 (49.1)
Sputum examination room	108 (85.0)	20 (15.7)	85 (66.9)	121 (95.3)	80 (66.1)	18 (14.9)	58 (47.9)
X-ray room	38 (29.9)	35 (27.6)	55 (43.3)	73 (57.5)	33 (45.2)	9 (12.3)	34 (46.5)

UVGI = Ultraviolet germicidal irradiation.

* daily opened in summer all day and daily opened in winter for short time.

** Quarterly tested or half-yearly tested.

### LTBI prevalence

**Figure 2:**
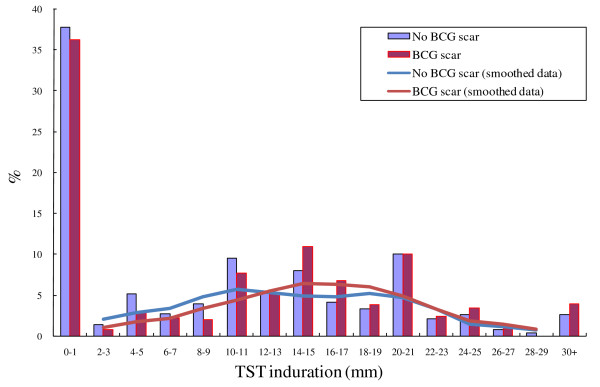
**Distribution of TST induration sizes among 2153 HCWs with BCG and without BCG vaccination scar**.

TST was employed in 2153 (94.1%) out of 2288 HCW in the 58 selected TB centers. Of the 135 non-respondents, 71 were working at a different location on the day the TST was applied, 30 were working at a different location on the day the TST was read, 20 were on holiday, and 34 decided to not participate. The mean age of the 2153 HCW was 37 (18-61) years old, 1163 (54.0%) were female, and 990 (45.0%) were male. Despite training, terminal-digit preference was visible at 10, 15, and 20 mm. An analysis of smoothed data indicated that there was no apparent bimodal distribution in HCW with or without BCG scar. Large TST indurations (≥ 15 mm) were more frequent in those with BCG scars (Figure 2). LTBI prevalence in those with and without BCG scar was 55.6% (432/777) and 49.0% (674/1376), respectively (OR = 1.3, 95%CI = 1.1-1.6). 36.7% (534/1455) of medical staff and 34.8% (243/698) of administrative/logistic staff had BCG scars (OR = 1.1, 95%CI = 0.9-1.3).

LTBI was associated with working at a TB center at the prefecture level, older age, longer duration of employment, presence of BCG scar, employment at a TB center with an inpatient ward, and job location within the TB center. Administrative/logistic staff and pharmacy staff had the lowest prevalence of LTBI (Table 2); LTBI prevalence in medical versus administrative/logistic staff was 55.7% (811/1455) and 42.3% (295/698), respectively (OR = 1.7, 95%CI = 1.4-2.1). Some factors associated with TST induration size ≥ 10 mm were similar for HCW with compared to those without a BCG vaccination scar, including age, job location, duration of employment, inpatient ward, and level.

**Table 2:** Risk factors of latent tuberculosis infection (LTBI) among 2153 health care workers

Variable		HCW N (%)	Induration ≥ 10 mm n (%)	Crude OR (95% CI)		Adjusted OR* (95% CI)	
Gender and smoking	Male, non-smoker	526 (24.4)	253 (48.1)	1			
	Male, smoker	464 (21.6)	244 (52.6)	1.2	(0.9-1.5)		
	Female	1163 (54.0)	609 (52.4)	1.2	(0.9-1.5)		
Age group (years)	18-29	580 (26.9)	229 (39.5)	1		1	
	30-39	740 (34.4)	399 (53.9)	1.8	(1.4-2.2)	1.6	(1.2-2.1)
	40-49	615 (28.6)	343 (55.8)	1.9	(1.5-2.4)	1.8	(1.3-2.5)
	≥ 50	218 (10.1)	135 (61.9)	2.5	(1.8-3.4)	2.6	(1.7-4.0)
BCG scar	No	1376 (63.9)	674 (49.0)	1		1	
	Yes	777 (36.1)	472 (55.6)	1.3	(1.1-1.6)	1.4	(1.1-1.7)
Income (Yuan/Year)	< 15000	1106 (51.4)	528 (47.7)	1			
	15000-19999	691 (32.1)	361 (52.2)	1.2	(1.0-1.5)		
	20000-29999	298 (13.8)	182 (61.1)	1.7	(1.3-2.2)		
	> 30000	58 (2.7)	35 (60.3)	1.7	(1.0-2.9)		
Education	Primary or middle school	63 (2.9)	28 (44.4)	1			
	Secondary school	976 (45.3)	450 (46.1)	1.1	(0.6-1.8)		
	Junior college	898 (41.7)	496 (55.2)	1.5	(0.9-2.6)		
	University or above	216 (10.1)	132 (61.1)	2.0	(1.1-3.5)		
Position	Junior	1270 (59.0)	594 (46.8)	1			
	Middle	697 (32.4)	414 (59.4)	1.7	(1.4-2.0)		
	Senior	186 (8.6)	98 (52.7)	1.3	(0.9-1.7)		
Job location	Administrative/logistic	698 (32.4)	295 (42.3)	1		1	
	TB outpatient clinic	609 (28.3)	333 (54.7)	1.7	(1.3-2.1)	1.9	(1.4-2.6)
	TB inpatient ward	165 (7.7)	101 (61.2)	2.2	(1.5-3.1)	1.3	(1.0-2.1)
	Supervision and monitoring	234 (10.9)	132 (56.4)	1.8	(1.3-2.4)	1.6	(1.2-2.3)
	Pharmacy	147 (6.8)	74 (50.3)	1.4	(1.0-2.0)	1.0	(0.7-1.6)
	X-ray department	122 (5.6)	78 (63.9)	2.4	(1.6-3.6)	1.8	(1.1-2.9)
	Laboratory	178 (8.3)	93 (52.2)	1.5	(1.1-2.1)	1.4	(0.9-2.0)
Duration of employment (years)	< 1	810 (37.6)	295 (36.4)	1		1	
	1-4	374 (17.4)	166 (44.4)	1.4	(1.1-1.8)	1.4	(1.0-1.9)
	5-9	323 (15.0)	188 (58.2)	2.4	(1.9-3.2)	2.3	(1.6-3.1)
	≥ 10	646 (30.0)	457 (70.7)	4.2	(3.4-5.3)	3.0	(2.2-4.1)
Inpatient ward	No	1094 (50.8)	436 (39.9)	1		1	
	Yes	1059 (49.2)	670 (63.3)	2.6	(2.2-3.1)	2.3	(1.9-2.9)
Size of clinic (No. of staff)	< 25	348 (16.2)	179 (51.4)	1			
	25-39	600 (27.9)	268 (44.7)	0.8	(0.6-0.9)		
	≥ 40	1205 (56.0)	659 (54.7)	1.1	(0.9-1.5)		
Level	County/District	1282 (59.6)	538 (42.0)	1		1	
	Prefecture	871 (40.4)	568 (65.2)	2.8	(2.3-3.3)	1.8	(1.0-3.5)

*Adjusted for all other factors included in the multivariate model.

HCW = health care worker; OR = odds ratio; CI = confidence interval; TB = tuberculosis.

### TB disease prevalence

In the 127 TB centers, all HCW gave consent to be screened. Among the 3759 HCW, 3746 (99.7%) were investigated for pulmonary TB disease, and the rest 13 (0.3%) were absent from work during the investigation. The mean age of the 3746 HCW was 37 (18-61) years old, 1937 (51.7%) were female, and 1809 (48.8%) were male. Among the 3746 HCW, 86 (2.3%) reported TB symptoms, 54 (1.4%) had an abnormal chest X-ray, and only 9 (0.2%) had both TB symptoms and an abnormal chest X-ray. Twenty (0.5%) cases of pulmonary TB were detected including 18 new cases and 2 retreatment cases, and 3 out of 20 cases were diagnosed as new smear-positive cases. The overall point prevalence was 5.3/1000; 6.7/1000 among medical staff and 2.5/1000 among administrative/logistic staff (OR = 2.7, 95%CI = 0.8-9.3). HCW with longer duration of employment seemed at increased risk of active TB; the TB prevalence was 3.7/1000 among HCW who worked for less than 5 years in the facility and 7.5/1000 among HCW who worked for ≥ 5 years in the facility (OR = 2.0, 95%CI = 0.8-4.9). The prevalence for HCW working with TB inpatients was 15.2/1000, which was much higher than in HCW working in any other job location (OR = 3.2, 95%CI = 1.1-11.1). There were only 9 female smokers out of 1937 (4.6/1000) female HCWs, so we combined gender and smoking into three subgroups: females, male non-smokers, and male smokers. TB prevalence in male smokers was 9.6/1000 and was higher than in male non-smokers (OR = 9.5, 95% = 1.2-75.9) (Table 3). There was no significant difference in TB disease prevalence by age, BCG scar, job location, and administrative level of the TB center.

**Table 3:** Tuberculosis prevalence in 3746 health care workers in Henan province, China

Item		No. of HCW	No. of TB	Prevalence (1/1000)
Gender and smoking*	Male, non-smoker	977	1	1.0
	Male, smoker	832	8	9.6
	Female	1937	11	5.7
Age group (years)	18-29	988	6	6.1
	30-39	1295	5	3.9
	40-49	1070	7	6.5
	≥ 50	393	2	5.1
BCG scar	No	2451	13	5.3
	Yes	1294	7	5.4
Job location	TB outpatient clinic	1187	9	7.6
	TB inpatient ward	197	3	15.2
	Supervision and monitoring	383	1	2.6
	Pharmacy	255	1	3.9
	X-ray	204	1	4.9
	Laboratory	305	2	6.6
	Administrative/logistic	1215	3	2.5
Duration of employment (years)	< 1	1383	5	3.6
	1-4	757	3	4.0
	5-9	613	6	9.8
	≥ 10	993	6	6.0
Level	Prefecture	869	5	5.8
	County/District	2877	15	5.2
TST induration (mm)^§, #^	0 - 9	1047	1	1.0
	10 - 19	687	6	8.7
	20 +	419	7	16.7

* Male smoker: OR = 9.5, 95%CI = 1.2-75.9; Female: OR = 5.6, 95%CI = 0.7-43.2.

§ in 2153 HCW with TST results including 14 TB cases.

# 10-19 compared to 0-9: OR = 9.2, 95%CI = 1.1-76.6; 20+ compared to 0-9: OR = 17.6, 95%CI = 2.2-143.5.

HCW = health care worker; TB = tuberculosis; TST = tuberculin skin test.

The identified TB patients had TST induration sizes that ranged from 6 mm to 50 mm, with a median of 20 mm. Only one patient had an induration < 10 mm. In the 2153 HCW with TST results, TB disease prevalence in HCW with TST induration < 10 mm was 1.0/1000, and in HCW with TST induration ≥ 10 mm was 11.8/1000 (OR = 12.4, 95%CI = 1.6-95.3). TB prevalence increased with larger TST induration sizes (Table 3).

## Discussion

Some aspects of TB infection control in TB centers as evaluated in this study appeared inadequate in Henan province of China. The prevalence of LTBI and TB disease among HCW was higher than observed in the national TB prevalence survey in 2000 [3]. The LTBI prevalence of 56% among medical staff was higher than the LTBI prevalence of 47% (using a cut-off point of 6 mm) in the general population group of 15 years or older from the survey in 2000. So even with a higher TST cut-off point, the estimated prevalence of LTBI amongst HCW was still higher than the prevalence in the general population group of 15 years or older in a survey conducted five years earlier. We observed a TB disease prevalence of 5.3/1000 among HCW compared to 3.0/1000 in the 15-59 year old age group in the prevalence survey. The age- and sex-stratified prevalence in the 2000 survey were applied to calculate the expected number of cases in 2005 in HCW. The calculation indicated 12 expected active TB cases but instead our study found 20 cases, which was much higher than estimated (RR = 1.67, 95%CI = 1.02-2.57).

Studies elsewhere showed varying LTBI rates amongst HCW [7,8,20,21,23]. Our results from Henan province showed high LTBI rates amongst HCW similar to other studies in low and middle income countries and compared well with an average LTBI prevalence of 54% among HCW [8]. The prevalence of LTBI among HCW in high-income countries was generally lower [24,25]. Our study results are also consistent with studies that show an increasing prevalence of LTBI with increasing age, work locations with higher exposure to TB patients (especially TB outpatient clinics, inpatient wards, and X-ray departments), and longer duration of employment at the health care facility [7,8,20,23]. In our study, working at a TB center at the prefecture level and presence of an inpatient ward at the TB center were associated with a higher LTBI prevalence among staff members. At prefecture level, all TB centers had TB clinics and inpatient wards. Patients with more severe TB disease likely attend a prefecture TB center for medical care, which may increase the exposure to TB at prefecture level. Although the association of male sex with increased *M. tuberculosis *infection risk has been reported [19], we did not observe this (OR = 1.1, 95% CI: 0.9-1.3). However, we did observe that male smokers had an increased prevalence of TB disease compared with non-smokers, as shown before [26].

This study suggests that nosocomial transmission of TB is an important occupational problem among HCW. The reduction of this risk should be a priority. Occupational contracted TB can lead to the loss of skilled workers, and this can adversely impact on health care services in the long term. Medical workers may also avoid working with TB centers due to the high risk of TB transmission to HCW. TB amongst HCW can have serious, and even fatal, consequences. This problem is particularly serious with MDR-TB strains. Hospitals have been shown to be important focal points of MDR-TB transmission, resulting in explosive outbreaks [27,28]. Implementation of effective TB infection control measures can promote awareness of the disease among HCW, and help in the adoption of good practices for diagnosis and treatment of TB.

Most of the TB centers in China are in older buildings, and therefore are not designed in line with TB infection control standards [10]. Therefore, we suggest beginning implementation of some simple yet highly effective interventions, such as fast identification of TB suspects, segregation of infectious TB patients, and education as well as training for HCW [10]. Administrative interventions that improve the current situation in health care facilities require minimal resources and can be easily implemented. Engineering controls such as exhaust ventilation, improved natural ventilation [29], and the use of UV lights are also cost-effective measures to minimize the risk of TB infection [10]. National TB control and public health authorities should focus on addressing nosocomial TB transmission as an integral part of the TB control program. HCW are essential in the fight against TB, and their health should be protected as well as that of patients. With the emergence of XDR-TB, the need to implement TB infection control measures has been reemphasized by the WHO and the Stop TB Partnership [30]. After the study results became available, TB centers in Henan province have strengthened training on TB infection control for HCW and paid more attention to ventilation systems and adequate UV light use.

There are several limitations to our study. The trained project staff filled in the checklists for their own centers, and therefore the staff might not have been completely honest about prevailing TB infection control practices in their own centers. Even with intensive training for TST nurses, the terminal-digit preference was visible at 10, 15, and 20 mm. The preference for cut-off point of 10 mm in TST results may have caused a slight overestimation of HCW with LTBI. Our results showed that people with BCG vaccination had higher prevalence of LTBI. BCG vaccination can also lead to positive results of the TST, thus we may have overestimated the LTBI rate in BCG vaccination group. However, a couple of international reviews have reported that the influence of BCG is relatively small in the adult population, especially ≥ 10 years after BCG vaccination [8,14]. Misclassification of BCG vaccination status may have occurred as not all of those vaccinated develop a scar. However, the presence of a BCG scar has been found to be a good indicator of BCG vaccination [31] and several studies reported that 95-100% people had a BCG scar after BCG vaccination [31-33] so misclassification should be limited. Exclusion of those with BCG scar did not affect results. LTBI or TB disease in a HCW may be due to infection acquired nosocomially or in the community. We were unable to differentiate between infections acquired in the community versus in the center, but LTBI prevalence in medical staff (56%) was significantly higher than that in administrative/logistic staff (42%) in the same TB center. This indicates that at least (((56-42)/56) =) 25% of LTBI in this setting could be attributed to nosocomial infection. We observed a TB disease prevalence that was much higher than observed in the 2000 survey. This may be explained by the fact that the 2000 survey used the chest fluoroscopy, which has a much lower sensitivity than the chest X-ray method, for detecting TB. Moreover, only three of the twenty TB disease cases were smear positive. It might be that we identified some TB patients at very early stage with tiny shadows at the chest X-ray, as the majority did not have any symptoms.

We are the first to report internationally on prevalence of TB infection and disease among Chinese HCW. As TB infection control was at a very early stage at the time of the study, the infection control situation in most areas of China was likely to be similar to Henan province. In a few MDR-TB project areas in China, the TB infection control policy as recommended by the WHO is now being implemented through the national TB program [10]. TB infection control is insufficient in most areas of China. With a high TB prevalence and limited resources, China focuses largely on case detection and treatment using the DOTS strategy. In some areas, even low-cost strategies to reduce TB transmission in health-care facilities are seldomly implemented. We strongly suggest that TB infection control strategies are rapidly implemented, with priority for provinces with high anti-TB drug resistance prevalence.

## Conclusion

TB infection control in TB centers in Henan province appeared to be inadequate and the prevalence of LTBI and TB disease among HCW was high. TB infection control practices in TB centers should be strengthened in China, including administrative measures, renovation of buildings, and the use of respirators and masks. Moreover, regular screening of HCW for TB disease and infection needs to be considered, offering preventive therapy to those with TST conversions.

## Competing interests

The authors declare that they have no competing interests.

## Authors' contributions

GXH participated in the design and coordination of the study, was responsible for data collection, analysis and interpretation of the data and drafted the manuscript. SH assisted in analysis and interpretation of the data, and assisted in drafting the manuscript. MJW assisted in analysis and interpretation of the data, and critically reviewed the manuscript. GJW participated in the design and coordination of the study, was responsible for data collection and interpretation of the data. SWM and DYZ were involved in the design and coordination of the study, was responsible for data collection and interpretation of the data. YLH was responsible for data collection, analysis and interpretation of the data. SCY was responsible for data analysis and interpretation of the data. MWB assisted in analysis and interpretation of the data and critically reviewed the manuscript. All authors reviewed and approved the manuscript.

## Pre-publication history

The pre-publication history for this paper can be accessed here:

http://www.biomedcentral.com/1471-2334/10/313/prepub
